# Effects of Low Body Mass Index and Smoking on All-cause Mortality among Middle-aged and Elderly Japanese

**DOI:** 10.2188/jea.12.40

**Published:** 2007-11-30

**Authors:** Motonobu Miyazaki, Akira Babazono, Toshiya Ishii, Takuya Sugie, Yoshito Momose, Mitsue Iwahashi, Hiroshi Une

**Affiliations:** 1 Department of Hygiene and Preventive Medicine, School of Medicine, Fukuoka University.; 2 Institute of Health Science, Kyushu University.; 3 Narita Airport Quarantine Station, Ministry of Health, Labor and Welfare.; 4 Department of Hygiene and Preventive Medicine, School of Medicine, Okayama University.

**Keywords:** body mass index, smoking, mortality, epidemiology, middle-aged and elderly Japanese

## Abstract

To investigate effects of low body mass index (BMI) and smoking on all-cause mortality among middle-aged and elderly Japanese, we conducted a community-based prospective study. A mail survey was conducted in 1987-1990 in four towns, western Japan. A cohort of 7,301 Japanese men and 8,825 Japanese women was followed up from the date of the mail survey to 1995 in three of the towns and 1998 in the fourth town. We investigated the effect of BMI and smoking on all-cause mortality by using Cox’s proportional hazards model. The relationship between BMI and all-cause mortality was a reverse J-shape with minimal mortality in 24≦BMI<26 in men and a U-shape with minimal mortality in 22≦BMI<24 in women, after adjusting for age and smoking. The lowest BMI category (BMI<20) had the highest all-cause mortality in men and also in women. Taking only never-smokers, the highest risk for all-cause mortality was observed in the lowest BMI category for men and for women. This does not seem to be explained by smoking and pre-existing diseases. More attention should be paid to persons with low BMI.

## INTRODUCTION

It has been widely recognized that obesity is a major health issue and increases the risk of cardiovascular disease^1^^-^^7^^)^. However, many epidemiological studies investigating the association between body mass index (BMI) and all-cause mortality have found a U-shaped relationship and observed an elevated risk of mortality in low BMI groups as well as in those with high BMI ones^2^^-^^9^^)^. Nakayama et al^6^^)^, in their Hisayama Study, reported that the lowest BMI category had the highest mortality risk among middle-aged and elderly persons. Strawbridge et al^10^^)^ insisted that more attention should be paid to serious health risks associated with low weight. Some researchers have suggested that the excess mortality of thin persons might be attributable to cigarette smoking and pre-existing diseases^2^^,^^4^^,^^11^^,^^12^^)^.

To clarify effects of low BMI and smoking on all-cause mortality, we examined relationships between BMI and all-cause mortality among smokers and never-smokers separately.

## MATERIALS AND METHODS

We conducted a mail survey in four towns in the western part of Japan during 1987-1990. This mail survey was performed in cooperation with a public health center and four local government offices in these towns. In the four towns, the population aged 40-69, which was identified from the municipal population registers, was 12,435 men and 14,627 women. We mailed a questionnaire including information on medical history, smoking habit, alcoholic drinking habit, and occupational history.

The follow-up period extended from the date of the mail survey to February 28th, 1995 in three of the towns and to November 30th, 1998 in one of the towns. We identified 742 emigrants through the municipal population registers, and confirmed all deaths from death certificates in three of the towns and from the municipal population registers in one of the towns.

BMI was defined as weight (kg) divided by the square of height (m). The subjects were divided into five categories of BMI as follows; BMI<20, 20≦BMI<22, 22≦BMI<24, 24≦BMI<26, and 26≦BMI, and classified according to the smoking status into never-smokers, ex-smokers and current smokers consuming <20 or 20≦ cigarettes daily.

We applied Cox’s proportional hazards model to investigate effects of BMI and smoking on all-cause mortality. The Statistical Analysis System (SAS) software package (SAS Institute, Cary, NC, USA) was used for data analysis. “Significant” indicates that 95% confidence intervals (CIs) do not include 1.00.

## RESULTS

### 1. Response rate

Table 1 shows response rates according to age. The overall response rate was 58.7% in men and 60.3% in women. The response rates increased with age in men and in women.

**Table 1.** Number of respondents and non-respondents according to age.

Age	Men			Women		
	
	Respondents	Non-respondents	Total	Respondents	Non-respondents	Total
40-49	2429 (50.4)	2389 (49.6)	4818 (100)	2628 (52.9)	2341 (47.1)	4969 (100)
50-59	2435 (58.2)	1752 (41.8)	4187 (100)	2996 (61.3)	1888 (38.7)	4884 (100)
60-69	2437 (71.0)	993 (29.0)	3430 (100)	3201 (67.1)	1573 (32.9)	4774 (100)

Total	7301 (58.7)	5134 (41.3)	12435 (100)	8825 (60.3)	5802 (39.7)	14627 (100)

% in parentheses

### 2. BMI and smoking status

The mean BMI was 22.8±2.8 (mean±SD) in men and 22.7±3.0 (mean±SD) in women. Table 2 shows the smoking status of the subjects by the five categories of BMI. The rates of current smoking were 55.4% in men and 9.9% in women. A rate decreased with BMI; from 65.6% in BMI<20 to 48.1% in 26≦BMI in men, but such a trend was not observed in women.

**Table 2.** Smoking status of subjects by five categories of body mass index.

[Men]					

Body mass index	Never-smokers	Ex-smokers	Current smokers		Total

			<20 cigarettes/day	≧20 cigarettes/day	
BMI<20	190 (16.7)	201 (17.6)	275 (24.1)	473 (41.5)	1139 (100)
20≦BMI<22	361 (19.9)	354 (19.5)	359 (19.8)	741 (40.8)	1815 (100)
22≦BMI<24	463 (22.6)	501 (24.4)	326 (15.9)	762 (37.1)	2052 (100)
24≦BMI<26	362 (25.5)	369 (26.0)	181 (12.8)	505 (35.6)	1417 (100)
26≦BMI	228 (26.0)	228 (26.0)	98 (11.2)	324 (36.9)	878 (100)

Total	1604 (22.0)	1653 (22.6)	1239 (17.0)	2805 (38.4)	7301 (100)

[Women]					

Body mass index	Never-smokers	Ex-smokers	Current smokers		Total

			<20 cigarettes/day	≧20 cigarettes/day	

BMI<20	1308 (85.0)	40 ( 2.6)	135 ( 8.8)	56 ( 3.6)	1539 (100)
20≦BMI<22	2008 (88.7)	48 ( 2.1)	130 ( 5.7)	77 ( 3.4)	2263 (100)
22≦BMI<24	2030 (88.8)	62 ( 2.7)	122 ( 5.3)	71 ( 3.1)	2285 (100)
24≦BMI<26	1419 (89.0)	40 ( 2.5)	80 ( 5.0)	55 ( 3.5)	1594 (100)
26≦BMI	946 (82.7)	50 ( 4.4)	74 ( 6.5)	74 ( 6.5)	1144 (100)

Total	7711 (87.4)	240 ( 2.7)	541 ( 6.1)	333 ( 3.8)	8825 (100)

% in parentheses

### 3. Hazard ratios of BMI and smoking for all-cause mortality

Table 3 shows hazard ratios of BMI and smoking for all-cause mortality. In men, the relationship between BMI and all-cause mortality was a reverse J-shape with minimal mortality among those of 24≦BMI<26; hazard ratios were 1.78 for BMI<20, 1.35 for 20≦BMI<22, 1.00 for 22≦BMI<24 (reference), 0.91 for 24≦BMI<26 and 1.11 for 26≦BMI. In women, the relationship was a U-shaped with minimal mortality among those of 22≦BMI<24; hazard ratios were 1.92 for BMI<20, 1.22 for 20≦BMI<22, 1.00 for 22≦BMI<24 (reference), 1.26 for 24≦BMI<26, and 1.56 for 26≦BMI.

**Table 3.** Hazard ratios of body mass index and smoking for all-cause mortality.

Variables	Men			Women		
	
	Number of subjects	Number of deceased	Hazard ratios (95%C.I.)	Number of subjects	Number of deceased	Hazard ratios (95%C.I.)
Age (10 year interval)			2.22 (1.96-2.53)			2.34 (1.97-2.79)
Body Mass Index						
BMI < 20	1139	130	1.78 (1.38-2.29)	1539	73	1.92 (1.35-2.72)
20≦BMI < 22	1815	141	1.35 (1.06-1.73)	2263	63	1.22 (0.85-1.76)
22≦BMI < 24	2052	116	1.00 (reference)	2285	55	1.00 (reference)
24≦BMI < 26	1417	68	0.91 (0.67-1.22)	1594	51	1.26 (0.86-1.84)
26≦BMI	878	47	1.11 (0.79-1.56)	1144	45	1.56 (1.05-2.31)
Smoking						
Never-smokers	1604	72	1.00 (reference)	7711	221	1.00 (reference)
Ex-smokers	1653	123	1.35 (1.01-1.81)	240	12	1.38 (0.77-2.47)
Current smokers						
< 20 cigarettes/day	1239	121	1.61 (1.20-2.16)	541	36	2.07 (1.45-2.95)
≧ 20 cigarettes/day	2805	186	1.44 (1.10-1.90)	233	18	2.02 (1.25-3.26)

Adjusted for variables in this table.

C.I.=Confidence interval

Hazard ratios of smoking for all-cause mortality were significantly high: 1.35 for ex-smokers, 1.61 for current smokers of<20 cigarettes/day and 1.44 for 20≦ cigarettes/day.

### 4. Hazard ratios of body mass index for all-cause mortality among ever-smokers and among never-smokers

As shown in Table 4 and Table 5, we examined relationships between BMI and all-cause mortality among never-smokers and among ever-smokers (ex-smokers + current smokers) separately.

**Table 4.** Odds ratios of body mass index for all-cause mortality among never-smokers.

Variables	Men			Women		
	
	Number of subjects	Number of deceased	Hazard ratios (95%C.I.)	Number of subjects	Number of deceased	Hazard ratios (95%C.I.)
Age (10 year interval)			2.85 (2.02-4.02)			2.29 (1.89-2.79)
Body Mass Index						
BMI < 20	190	21	3.30 (1.65-6.60)	1308	61	2.33 (1.57-3.45)
20≦BMI < 22	361	21	2.10 (1.05-4.20)	2008	48	1.27 (0.81-1.84)
22≦BMI < 24	463	13	1.00 (reference)	2030	42	1.00 (reference)
24≦BMI < 26	362	9	0.96 (0.41-2.25)	1419	39	1.27 (0.82-1.96)
26≦BMI	228	8	1.37 (0.57-3.31)	946	31	1.57 (0.99-2.50)

Adjusted for variables in this table.

C.I.=Confidence interval

**Table 5.** Odds ratios of body mass index for all-cause mortality among ever-smokers.

Variables	Men			Women		
	
	Number of subjects	Number of deceased	Hazard ratios (95%C.I.)	Number of subjects	Number of deceased	Hazard ratios (95%C.I.)
Age (10 year interval)			2.13 (1.86-2.44)			2.56 (1.71-3.82)
Body Mass Index						
BMI < 20	949	109	1.61 (1.23-2.11)	231	12	0.93 (0.42-2.04)
20≦BMI < 22	1454	120	1.27 (0.97-1.65)	255	15	1.25 (0.59-2.62)
22≦BMI < 24	1589	103	1.00 (Reference)	255	13	1.00 (Reference)
24≦BMI < 26	1055	59	0.91 (0.66-1.25)	175	12	1.22 (0.56-2.67)
26≦BMI	650	39	1.09 (0.75-1.55)	198	14	1.46 (0.68-3.11)

Adjusted for variables in this table.

C.I.=Confidence interval

In never-smokers, relationships between BMI and all-cause mortality were a reverse J shape among men and a U-shape among women. Hazard ratios for the group of BMI<20 were 3.30 for men and 2.33 for women, compared with the group of 22≦BMI<24 as a reference. These were statistically significant and the highest among the five BMI categories.

In ever-smokers, men also showed a reverse J shaped relationship between BMI and all-cause mortality. Among women, on the other hand, small numbers prevented there being a clear relationship between BMI and all-cause mortality. Hazard ratios for the group of BMI<20 were 1.61 for men, and this was statistically significant and the highest among the five BMI categories. However, high hazard ratios for the group of BMI<20 were not observed among women.

## DISCUSSION

Many epidemiological studies have been conducted to examine relationships between BMI and mortality in European countries and the US where obesity is a serious issue, and in other countries^2^^-^^13^^)^. Although the curve expressing the relationships between BMI and all-cause mortality varied according to the distributions of age and BMI of subjects studied^14^^)^, BMI showed a U-shaped relationship with all-cause mortality with high rates in both thin persons and obese persons in many of these studies^2^^-^^9^^)^. Some papers indicated that the increased mortality in high BMI is mainly attributable to cardiovascular disease^2^^-^^7^^,^^13^^)^ and, in low BMI, to infectious diseases, and respiratory diseases, and smoking-related cancer^2^^,^^3^^,^^6^^,^^11^^,^^13^^)^.

In Japan, there have been few prospective studies to evaluate the relationship between BMI and mortality^6^^,^^14^^)^. Tsukamoto et al^9^^)^ reported a U-shaped relationship between body weight and mortality with the nadir near the average weight for both men and women in a large life insurance cohort study in Japan, though they did not control for smoking, which is the most important risk factor for health, in their analysis.

Our results showed a reverse J-shaped relationship between BMI and all-cause mortality for men and a U-shaped relationship for women. The lowest rate for men was observed in the group of 24≦BMI<26, and for women, in the group of 22≦BMI<24.

Although our study did not reveal a U-shaped relationship between BMI and all-cause mortality for men, this may be because our study included few subjects with a BMI over 30 fulfilling the WHO criterion for obesity. Strawbridge et al^10^^)^ reported that in the US, a mortality risk was only modestly elevated for persons with BMIs of 25.0 through 29.9, but rose sharply with a BMI of more than 30. In the US, 12.0% of men and 15.0% of women had a BMI of 30 or more, but in our study, the prevalence of obesity (30≦BMI) was only 0.5% in men and 1.1% in women in our study. If our study had included sufficient subjects with BMIs of 28-30 and of more than 30, it is likely that it would have been a U-shaped relationship between BMI and all-cause mortality for men.

Tokunaga et al^16^^)^ indicated that the value of BMI associated with the lowest morbidity was 22.2 in men and 21.9 in women, and then suggested that the ideal BMI of Japanese is 22 in the 30-59 aged groups. However, minimum mortality occurred at BMI levels higher than 22 for men in our study, and also in many other epidemiological studies conducted to clarify the relationship between BMI and all-cause mortality^6^^,^^10^^,^^11^^,^^13^^,^^17^^)^.

In our study, the BMI level at which a minimum mortality occurs was higher in men than in women. If women have the same BMI as men, they tend to have more body fat than men. The ideal body weight and criteria of obesity should be defined separately for men and for women.

Thin persons had a high risk for all-cause mortality in both sexes in our study as well as in many other studies^2^^-^^12^^)^. Seidell et al^11^^)^ indicated that smoking was inversely associated with BMI levels. Our data are consistent with this result in men. A high mortality among thin persons was found to be partly attributable to smoking and pre-existing diseases^2^^,^^4^^,^^11^^,^^12^^)^. Because smoking is the most important risk factor for health and is more prevalent in the low BMI categories, we investigated the association between BMI and all-cause mortality among never-smokers and ever-smokers separately. The lowest BMI category also had the highest risk for all-cause mortality among never-smokers in men and in women.

To investigate the possibility that the earliest deaths might be due to pre-existing diseases, we excluded the first two years of follow-up and examined the association between BMI and all-cause mortality. However, hazard ratios remained highest in the lowest BMI category for men and for women. Hypertension and glycosuria were more common in the four groups of 20≦BMI than in the group of BMI<20 among men and among women in this study (data not shown). Therefore, smoking and the presence of pre-existing diseases do not explain this high risk for all-cause mortality in thin persons. Our study suggests that thinness is associated with a greatly increased risk for all-cause mortality among middle-aged and elderly persons, and therefore, as indicated by Strawbridge et al^10^^)^, more attention must be given to the health risk of thinness, as well as those of obesity.

Limitation of our study must be considered. Weight, height, and smoking status were self-reported. However, the distribution of BMI and smoking status in our subjects were comparable to those reported in the 1990 Japanese Nutrition Survey^19^^)^. Self-reported weight and height were found to be highly correlated with measured weight and height, though there was a small level of error^20^^,^^21^^)^.

Although we also mentioned in our previous paper^22^^)^ that the response rate in this study was relatively low, we could not clarify whether this caused a bias in the results.

In conclusions, our study suggests that thinness is associated with a greatly increased risk for all-cause mortality among middle-aged and elderly Japanese. This does not seem to be explained by smoking and pre-existing diseases.
